# Transcriptome wide analyses reveal intraspecific diversity in thermal stress responses of a dominant habitat‐forming species

**DOI:** 10.1038/s41598-023-32654-w

**Published:** 2023-04-06

**Authors:** Katy R. Nicastro, Gareth A. Pearson, Xana Ramos, Vasco Pearson, Christopher D. McQuaid, Gerardo I. Zardi

**Affiliations:** 1 CNRS, Univ. Littoral Côte d’Opale, UMR 8187 – LOG – Laboratoire d’Océanologie et de Géosciences, Univ. Lille, 59000 Lille, France; 2 CCMAR-CIMAR – Associated Laboratory, University of Algarve, Campus de Gambelas, 8005-139 Faro, Portugal; 3 Department of Zoology and Entomology, Rhodes University, Grahamstown, 6140 South Africa; 4 Department of Mathematics, Instituto Superior Técnico, 1049-001 Lisbon, Portugal; 5 UNICAEN, Laboratoire Biologie des Organismes et Ecosystèmes Aquatiques, UMR 8067 BOREA (CNRS, MNHN, UPMC, UCBN, IRD-207), Normandie Université, CS 14032, 14000 Caen, France

**Keywords:** Ecology, Genetics, Physiology

## Abstract

The impact of climate change on biodiversity has stimulated the need to understand environmental stress responses, particularly for ecosystem engineers whose responses to climate affect large numbers of associated organisms. Distinct species differ substantially in their resilience to thermal stress but there are also within-species variations in thermal tolerance for which the molecular mechanisms underpinning such variation remain largely unclear. Intertidal mussels are well-known for their role as ecosystem engineers. First, we exposed two genetic lineages of the intertidal mussel *Perna perna* to heat stress treatments in air and water. Next, we ran a high throughput RNA sequencing experiment to identify differences in gene expression between the thermally resilient eastern lineage and the thermally sensitive western lineage. We highlight different thermal tolerances that concord with their distributional ranges. Critically, we also identified lineage-specific patterns of gene expression under heat stress and revealed intraspecific differences in the underlying transcriptional pathways in response to warmer temperatures that are potentially linked to the within-species differences in thermal tolerance. Beyond the species, we show how unravelling within-species variability in mechanistic responses to heat stress promotes a better understanding of global evolutionary trajectories of the species as a whole in response to changing climate.

## Introduction

Living organisms generally need to maintain a relatively constant internal environment in order to grow and survive. Even within the same ecosystem, species have evolved different ways of maintaining homeostasis in the face of fluctuating and often harsh environmental conditions. Outside a given range of environmental conditions, a species’ physiology will be impaired and these limits, together with biotic interactions and the ability to colonise a region, will dictate the species’ distributional limits. Recently, the response of species to environmental stressors has received renewed attention because of the implications for understanding ecosystem vulnerability to climate change and declines in global biodiversity.

Species with wide distributions often experience a broad range of variable environmental conditions^1,2^. For instance, the intensity, frequency and duration of thermal stress may vary significantly across a species’ geographical ranges e.g.,^3^. Consequently, distinct populations may exhibit diverse thermal resilience resulting from phenotypic plasticity or adaptation^4,5^. In turn, discrete thermal biology matching within genetically structured populations may contribute to the establishment or maintenance of genetic divergence e.g.,^6,7^. Understanding whether distinct components of biodiversity below the species level respond homogeneously to temperature across their geographical range is of great conservation relevance because it facilitates effective and targeted management actions^8,9^. While studies investigating the increasing pressure imposed by current warming trends generally consider species as physiologically homogenous units, assessing intraspecific diversity in performance is a fundamental prerequisite to fully understanding the adaptive potential of the species as a whole e.g.,^5,10–12^.

Current high-throughput molecular approaches allow us to measure the expression of thousands of genes and proteins simultaneously and this has broadened our understanding of the molecular mechanisms underlying thermal stress responses e.g.,^13^, especially in model organisms e.g.,^14,15^. Stress responses include the expression of a series of evolutionarily conserved stress-responsive genes^16^ such as those controlling protein folding, degradation and repair, cell cycles, DNA and chromatin stabilization, and energy metabolism^17,18^. The genomic basis underlying different thermal tolerances below the species level are only just beginning to be described. In an age when changing climate is profoundly affecting the abundance and distribution of life on Earth, mitigating or managing the threat of climate change to global biodiversity and ecosystem services requires an understanding of the fundamental molecular mechanisms conferring resilience to thermal stress to particular portions of a species’ genetic pool.

**Figure 1:**
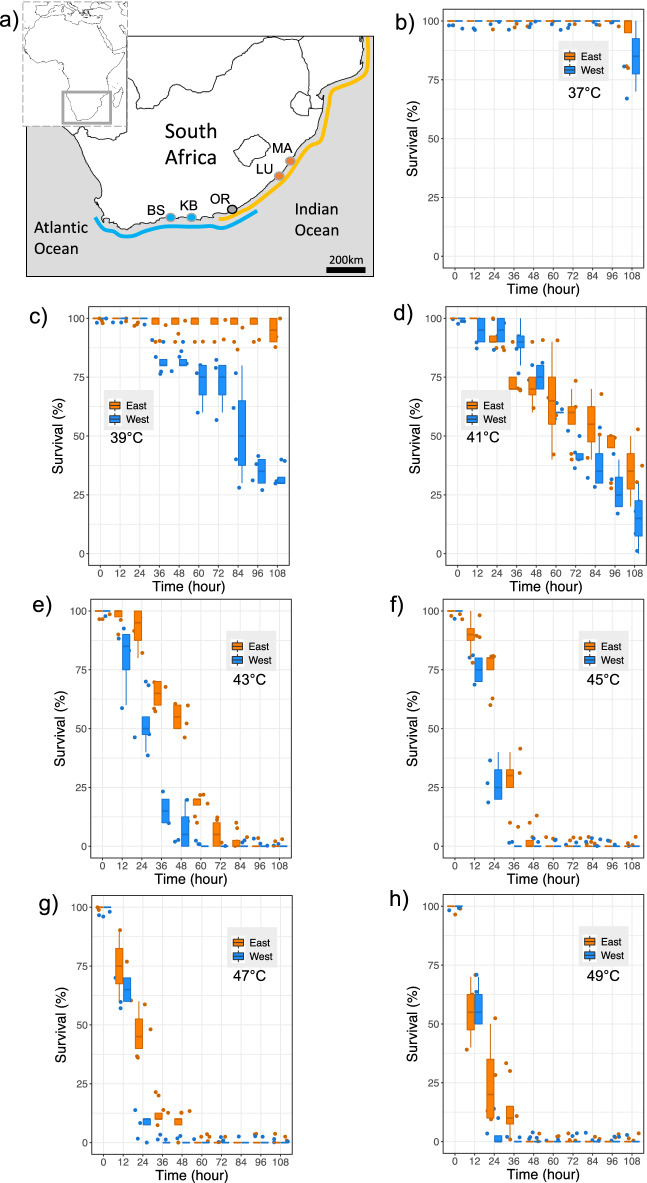
Map and survival rates in air. (**a**) Map showing the collection sites and the distribution of *Perna perna* genetic lineages in South Africa^20^. Survival rates in air at (**b**) 37 °C, (**c**) 39 °C, (**d**) 41 °C, (**e**) 43 °C, (**f**) 45 °C, (**g**) 47 °C, (**h**) 49 °C. Margate MA, Lusikisiki LU, Old Womans’ River OR, Keurboomstrand KB, Brenton-on-Sea BS. Survival curves between lineages were significantly different at 39 °C, 43 °C, 45 °C, 47 °C (long rank test; see Fig. S1). Maps were generated using SimpleMapp (https://www.simplemappr.net/#tabs=0).

Here, we investigate the transcriptional responses to heat stress (in air and water) of two geographically contiguous, but genetically distinct lineages of the intertidal mussel *Perna perna* along South African shores. *P. perna* is a key ecosystem engineer distributed widely around the world along warm water shores. In southern Africa, it dominates intertidal habitats in the sub-tropical and warm-temperate bioregions (Fig. 1a), but is absent from the cold water, west coast of South Africa and southern Namibia. Along the south east coast of South Africa, at the warm-temperate/subtropical biogeographic transition, analyses of mitochondrial DNA (mtDNA) and nuclear (ITS) sequence data and microsatellites, have revealed a sharp phylogeographic break between a western (Namibian coast and south coast of South Africa) and an eastern lineage. On the southeast coast, the distributions of the two lineages overlap over a distance of approximately 200 km^19^. Genetic analyses indicate a non-sister relationship for the two lineages that is explained by an initial Indo-Pacific origin followed by dispersal into the Mediterranean and the Atlantic via the Tethys seaway and recent secondary contact after independent southward expansion along the western and eastern coasts of the African continent^20^. Reciprocal field transplantation, oceanographic drifters, estimated directionality of gene flow and dispersal simulations show that phylogeographic divergence is maintained by environmental selective pressures and the influence of oceanographic dynamics on larval dispersal^7,21^.

In ecological contexts, comparative transcriptomic analyses can contribute to our understanding of the molecular basis of intraspecific variation (both plastic and locally adapted) in ecologically critical traits and the identification of genes of potential adaptive significance under variable environmental and climatic pressures. The aim of the study was to examine the nature of transcriptomic differences underlying thermal biology and local adaptation using the well-known within-species, genetic differentiation of *P. perna* as a model system.

## Methods

### Sampling and preparation of transplants

The study comprised two sets of common garden laboratory experiments, one of which examined the resilience of the two lineages to a range of temperature conditions in both air and water, and a second that was designed to provide tissue samples for the analysis of gene expression in the two lineages under thermal stress, again in air and in water.

For each set of experiments and for each genetic lineage, mussels were collected from two sites approximately 50–100 km apart (Fig. 1): Lusikisiki (31° 28′ 22.7″ S 29° 44′ 01.5″ E) and Margate (30° 51′ 57.2″ S 30° 22′ 19.6″ E) for the eastern lineage and Brenton-on-Sea (34° 04′ 28.4″ S 23° 01′ 14.2″ E) and Keurboomstrand (34° 00′ 17.6″ S 23° 27′ 18.8″ E) for the western lineage. Critically, to exclude potential biases triggered by differences in reproductive status between the two lineages, all mussels were sampled and transplanted in November. Numerous studies, over distinct years, have shown that, although the reproductive cycles of the two *P. perna* lineages are not always synchronized throughout the year, in November, eastern and western *P. perna* populations show the lowest values of gonadosomatic indexes following spawning^22–25^. Further, maximum values of gonadosomatic indices of the two lineages are not significantly different^22^ and references therein, suggesting that eastern and western mussels invest similar amounts of metabolic energy for reproduction.

Specimens (3.5–4.5 cm in shell length) were carefully collected from intertidal rocky shores in the mid mussel zone and brought to the laboratory within 8 h of collection. Mussels were placed in aquaria at 18 °C (average sea temperature conditions in the overlapping zone of the two lineages) with a 12 h light:dark cycle with frequent seawater changes (24–48 h) and constant aeration with air stones (hereafter referred to as acclimation conditions). Within 48 h, mussels were translocated to a field site on the south coast within the overlap region where the two lineages naturally co-occur (Old Womans’ River 33° 29′ 0.24''S 27° 8′ 57.048''E). There they were secured by screwed metal quadrats and mesh (size 0.25 cm) and kept in situ for one month. Mussels were then returned to the laboratory and maintained under acclimation conditions for an additional week before running the experiments to eliminate any stress induced by removal from the field.

### Resilience of the two lineages to heat stress

#### Laboratory experiment set-up

Trials were run using temperatures of 25, 27 and 31 °C for stress in water and 37, 39, 41, 43, 45, 47 and 49 °C for stress in air. These are extreme austral summer air and water temperatures (recorded in situ and supported by satellite data) experienced by mussels in the region where the distributions of the two linages overlap^21,26^. It is important to note that maximum air and water temperatures are not necessarily synchronised and can be markedly uncoupled. While air temperature is largely dictated by seasonal dynamics (i.e., higher temperatures during summer), water temperature is also strongly influenced by the Agulhas Current^25-2^. This warm current is about 60 to 100 km wide and flows to the southwest along the eastern and southern seaboard of South Africa (from 27° S to 40° S) at rates of 10 to 20 km day^−1^, following the 200 m isobath of the continental shelf from Maputo in Mozambique to the tip of the Agulhas Bank in South Africa^27^. The inshore thermal front of this current usually lies 14–38 km offshore, but it can flow onto the coast at 0–1 km offshore^27^ allowing sea water temperatures to deviate from normal seasonality and be warmer in winter than summer. Wind- and topographically-driven summer upwelling of cold bottom water reinforces this effect. For each lineage, 4 groups of ten mussels were used in each temperature trial. Percentage survival was verified at the end of each stress cycle. Gaping mussels that failed to respond by shell closure to physical stimulus after the recovery period were recorded as dead and discarded. The experiment ended after 186 h and 180 h for water and air stress respectively.

The air stress experiment was designed to simulate aerial exposure during low tide, with 4 h of stress and 8 h of recovery in water under acclimation conditions^7^. Relative humidity in the chamber during air stress was maintained between 50 and 60% to simulate relative humidity values in the field^28^.

For water stress, temperature was increased gradually from 18 °C (acclimation temperature) at a rate of 9 °C/h until the treatment temperature was reached. Animals were then exposed to the experimental temperature for 4 h followed by 8 h of recovery in water under acclimation conditions.

#### Data analyses

For each stress (air or water heat stress) and each temperature, survival data was used in a Kaplan–Meier Method to predict the probability that mussels belonging to each lineage would survive past time t and obtain an estimated survival probability as a function of individual characteristics^29^. For the purposes of the analysis, mussels that survived for the entire duration of the experiment were marked as “censored.” Mussels that died were marked as “event”. The survival probabilities for each lineage were then compared using a Log Rank Test, a non-parametric analysis that uses chi-square statistics to test for differences between survival curves and assumes equal accuracy data at a given time.

### Comparative gene expression under heat stress

#### Laboratory experiment set-up

For the air and water thermal stress treatments, mussels from each lineage/site were subjected to a treatment temperature of 40 °C in air or in 30 °C in water. These are sublethal temperatures consistent with maximum air and water temperatures recorded in situ during extreme high temperature events experienced by the species in the region where the distributions of the two lineages overlap^21,26^. Temperatures were raised gradually from the acclimation temperature over a period of 3 h at a rate of 9 °C/h until the treatment temperature was reached and then placed back in acclimation conditions for a 2 h recovery period before processing for RNA-sequencing. Control animals were kept in aquaria under acclimation conditions and otherwise treated identically. For the air treatment, relative humidity was maintained between 50 and 60%.

#### RNA isolation and Illumina sequencing

From each mussel (n = 2 from each site, giving n = 4 per lineage), mantle tissue was dissected and preserved in RNAlater. RNA-seq library preparation was done using a total of 25 mg of tissue from each mussel in 1 ml of RNAlater. Only males were used to account for putative sex differences in expression. To reduce the potential for increased variance in gene expression associated with variation in gonad development, the sexual cycle of each individual was confirmed as being similar by visual inspection of the gonads following dissection^30^. The quality of extracted RNA was examined by denaturing electrophoresis on 1.2% agarose gels; concentration and integrity were determined using the Experion Automated Electrophoresis System (Bio-Rad). Total RNA from 24 experimental samples (3 treatments × 2 lineages × 2 sites × 2 replicates/site) was used for library preparation and RNA-seq (HiSeq 2000; 100 bp PE reads). Clean full length reads from the service provider (BGI Tech Solutions, Hong Kong) were verified with FastQC (https://www.bioinformatics.babraham.ac.uk/projects/fastqc/) before downstream processing.

#### De novo* transcriptome assembly and annotation*

Reads from all samples were assembled de novo using rnaSpades^31^ with default parameters. In silico quality assessment of the raw transcriptome was performed with Transrate^32^ and “good” transcripts were retained (estimated from read pair alignment statistics, accuracy of base alignments and coverage distribution assessment; see https://hibberdlab.com/transrate/metrics.html for details). Open reading frame (ORF) prediction was performed on this set of transcripts using FragGeneScan^33^, and potential ORFs were screened by local Diamond Blastx^34^ against NCBI nr “Metazoa” proteins with an e-value cut off of 1e−10. Finally, transcripts with a significant hit were clustered at 97% nucleotide identity to collapse potentially duplicated ORFs VSearch^35^. The resulting reference transcriptome was annotated and used for subsequent phylogenetic, mapping and gene expression analyses^36^; Metazoa orthologue set using the gVolante online server (https://gvolante.riken.jp/). Functional annotation was performed against the UniRef90 database (https://www.uniprot.org/) using Diamond Blastx, e-value cut off 1e−10, retrieving linked Gene Ontology (GO), InterPro, and Pfam annotations for mapped accessions. Transcript mapping to Kyoto Encyclopedia of Genes and Genome (KEGG) orthologues was carried out via the KAAS-KEGG Automatic Annotation Server (https://www.genome.jp/kegg/kaas/).

#### Gene expression and enrichment analysis

High quality sample reads were mapped onto the reference transcriptome using the RSEM v1.2.31 wrapper script^37^ and Bowtie2 v2.4.2^38^. Expected count data were analysed in R using Bioconductor v3.12, edgeR^39^ and limma^40^. After preliminary analyses showed no effect of location nested within each lineage (“East” and “West”), samples from locations within each lineage were merged and differential expression (DE) was analysed between lineage-specific control groups and each thermal stress treatment (i.e., “WATER” and “AIR”). Graphical representations of the results were produced using R/Bioconductor packages ggplot2^41^, ComplexHeatMaps^42^, UpSetR^43^ and mixOmics^44^.

Gene set enrichment was based on adaptive clustering of GO terms^45^ and Mann–Whitney U tests^46^ on ranked signed log *P* values. Tests were performed using the GO_MWU R package as recommended^47^; https://github.com/z0on/GO_MWU.

#### Gene co-expression network analysis

Groups (or modules) of co-regulated genes were identified using weighted gene correlation network analysis WGCNA^48^. The input gene set was 5995 DE transcripts with adjusted (Benjamini-Hochberg) *P* value < 0.1 in any of our contrasts (i.e., between controls and treatments within lineage, or between lineages within treatment). This approach, rather than using the entire gene set, was chosen to reduce the computational burden without qualitatively affecting the expression modules identified. An unsigned co-expression network was built using a soft thresholding power of 8 (lowest value returning a scale-free topology fit index ≈ 0.9). Clustering of the topological overlap matrix (TOM) identified groups of highly co-expressed gene modules. Highly correlated modules were further merged based on the correlation of their eigengenes (> 0.75). Module-trait relationships were then examined by correlating module eigengenes with the phenotypic state of treatment and control samples for each lineage. Finally, clusters of highly co-expressed genes, based on the quantitative measure of their module membership and their gene significance (gene to trait correlation) were identified within modules as being associated with a particular trait (condition). Traits were treated as binary variables.

#### Phylogenetic analysis

The phylogenetic relationships among the samples were explored using 407 complete and unduplicated single-copy orthologues (≥ 501 bp) identified from BUSCO. Transcript *.fasta* sequences for BUSCO orthologues were extracted for each sample from sorted *.bam* files after phasing (samtools phase) and variant calling (bcftools mpileup). Allele sequences were obtained from the resulting *.vcf* files with vcfutils.pl and seqkit fq2fa, aligned with mafft and trimmed with Gblocks^49^ in TranslatorX^50^ using a custom python script. Locus alignments (see Dryad in data availability statement) were concatenated in a supermatrix of “pseudoalleles” and analysed by maximum likelihood (ML) in IQ-TREE 2^51^. The alignment was partitioned by fitting a separate evolutionary model of sequence evolution for each gene/ locus with ModelFinder, chosen based on BIC score. Gene and site concordance factor (CF) analysis, which estimates the percentage of gene trees (gCF) or sites (sCF) which agree with the consensus “species” tree from the partitioned ML supermatrix analysis, was also performed in IQ-TREE 2 following Minh et al.^51^ and the recommendations at http://www.iqtree.org/doc/. Replicate runs of the ML analysis (n = 10) were performed to assess the robustness of the topology, and nodal support of recovered relationships was generated using 1000 generated replicates for ultra-fast bootstraps.

## Results

### The two lineages show different resistance to thermal stress

Overall, in air, survival rates of mussels decreased at higher temperatures and differed between lineages, with the eastern lineage being more tolerant of high temperatures (Fig. 1b–h). The two survival curves were significantly different at 39 °C, 43 °C, 45 °C, 47 °C (long rank test; χ^2^ = 36.964, *P* = 0.001 at 39 °C; χ^2^ = 30.086, *P* = 0.001 at 43 °C; χ^2^ = 21.840, *P* = 0.001 at 45 °C and χ^2^ = 12.076, *P* = 0.001 at 47 °C; Fig. S1).

**Figure 2:**
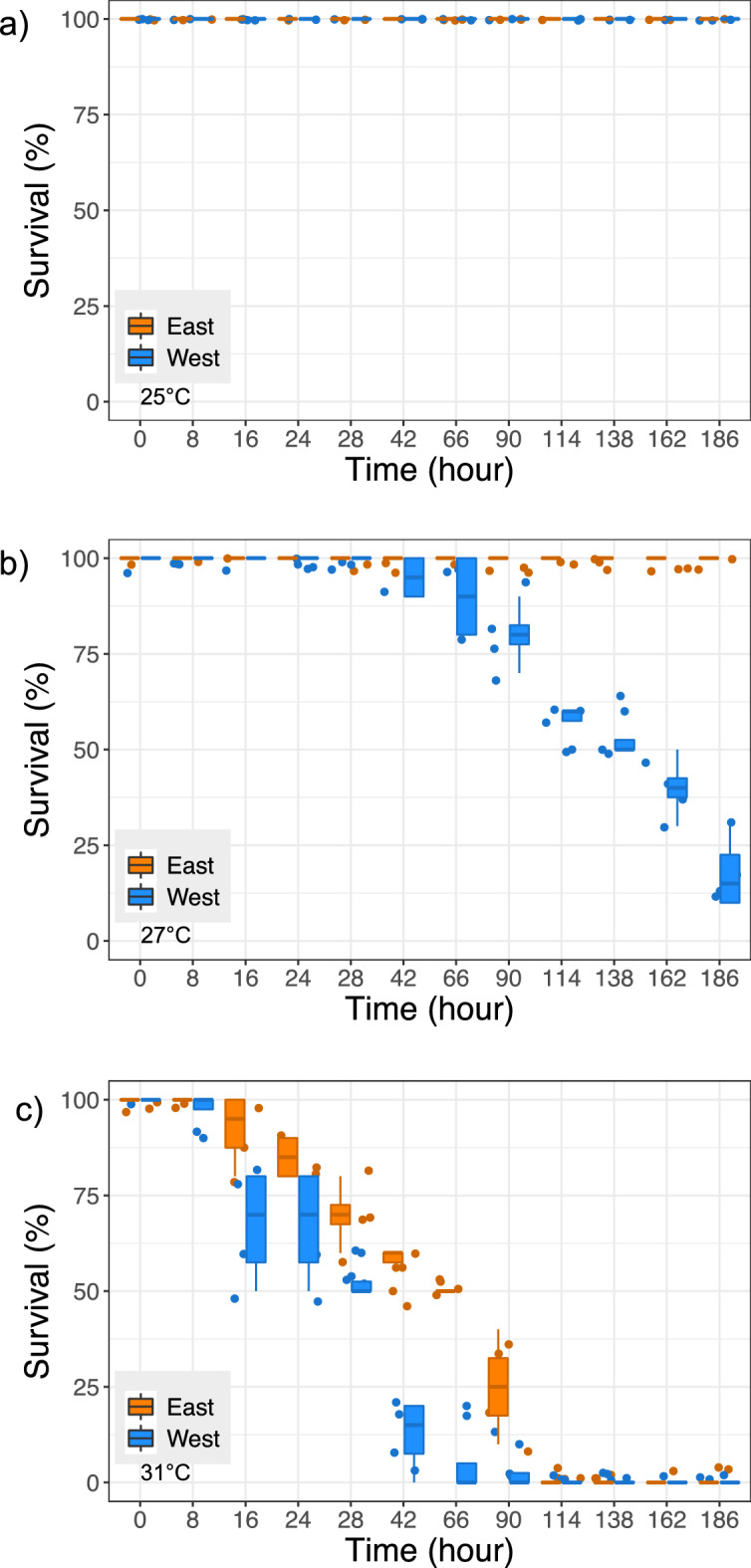
Survival rates in water at (**a**) 25 °C, (**b**) 27 °C, (**c**) 31 °C. Survival curves between lineages were significantly different at 27 °C and at 31 °C (long rank test; see Fig. 2S).

When subjected to thermal stress in water, mussel mortality increased with temperature and higher survival rates were recorded for eastern than western lineage mussels (Fig. 2). The two survival curves were significantly different at 27 °C and 31 °C (long rank test; χ^2^ = 57.801, *P* = 0.001 at 27 °C and χ^2^ = 18.447, *P* = 0.001 at 31 °C; Fig. S2).

#### Western and Eastern samples of Perna perna are phylogenetically distinct.

**Figure 3:**
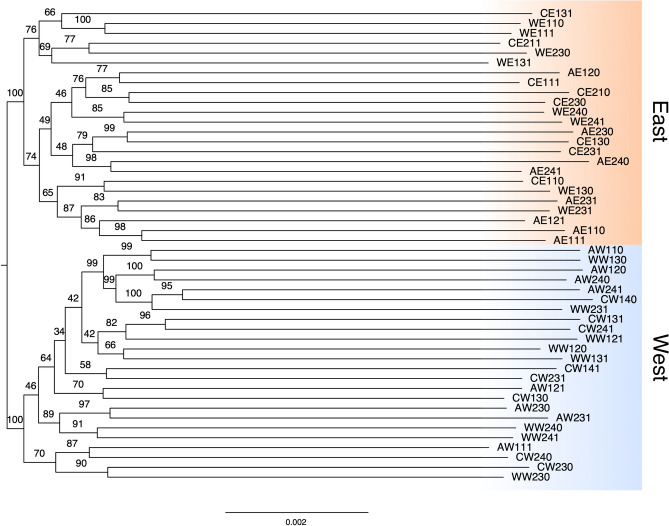
Maximum likelihood multilocus phylogenetic tree of the sample alleles used in this experiment. The tree was built from a concatenated supermatrix of aligned protein coding regions (434,025 sites) derived from 407 BUSCO single-copy orthologues. Ultrafast bootstrap values (1000 replicates) are indicated on branches. CE = Control for Eastern lineage, WE = Water for Eastern lineage, AE = Air for Eastern lineage, CW = Control for Wastern lineage, WW = Water for Western lineage, AW = Air for Western lineage.

Maximum likelihood analysis of 407 protein coding loci clearly showed that the samples used in these experiments were derived from each of these distinct lineages, which formed fully supported clades on the tree (Fig. 3). Branches supporting the Eastern and Western clades are very short in comparison with the divergence between individual (pseudo)alleles. Concordance factor (CF) analysis found no “decisive” gene trees (gCF = 0) and only a small excess of sites (sCF = 38.4%) supporting these branches (Fig. S3). The results are therefore consistent with distinct phylogroups with extensive incomplete lineage sorting (ILS) and support a proposed recent vicariant split between the lineages Cunha et al.^20^.

#### Transcriptome sequencing and gene expression profiles

The de novo assembly of 360.9M PE reads (72 Gb) resulted in 489,091 contigs, with 91.4% of reads mapped back by Transrate (aligned and quantified with SNAP and Salmon, respectively). The 81% of contigs (396,420) classified as “good” resulted in 307,564 potential ORFs subsequently identified by FragGeneScan. This resulted in a final reference containing 48,694 re-clustered contigs (28,817 unique accessions) with Blastx support against Metazoan proteins in the NCBI nr database. BUSCO analysis against 978 Metazoa single-copy orthologues indicated that the reference transcriptome was 95.8% complete (99.3% complete + partial sequences), with a duplication rate of 1.1 contigs/orthologue. These results therefore indicate a very complete reference with a low rate of redundancy/duplication.

**Figure 4:**
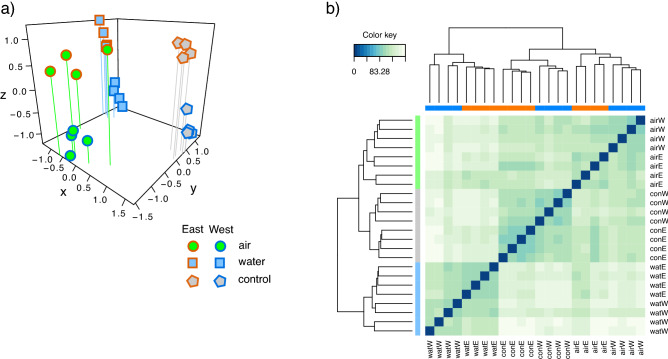
Sample clustering. Multidimensional scaling of samples (15,725 transcripts passing edgeR expression filters) in 3 dimensions coloured by treatment (**a**) and lineage (**b**). Sample distances for the same data (**c**) with row annotations indicating treatment (green = “AIR”, grey = “CONTROL” and light blue = “WATER”) and column annotations indicating lineage (blue = “West” and orange = “East”).

After sample mapping a total of 15,725 transcripts passed expression filters for analysis in edgeR-limma (Table S1). Ordination by multidimensional scaling (Fig. 4a,b) indicated that samples were primarily separated according to treatment (x, y axes) and secondarily by lineage on the third (z) axis. Sample distances (Fig. 4c) confirmed the same pattern, showing that CONTROL and AIR samples clustered more closely together compared to samples treated in WATER. Clustering also indicated considerable inter-individual distances, and incomplete lineage differentiation in response to AIR and WATER (Fig. 4c).

#### Thermal stress induced a greater transcriptional response in water than in air

**Figure 5:**
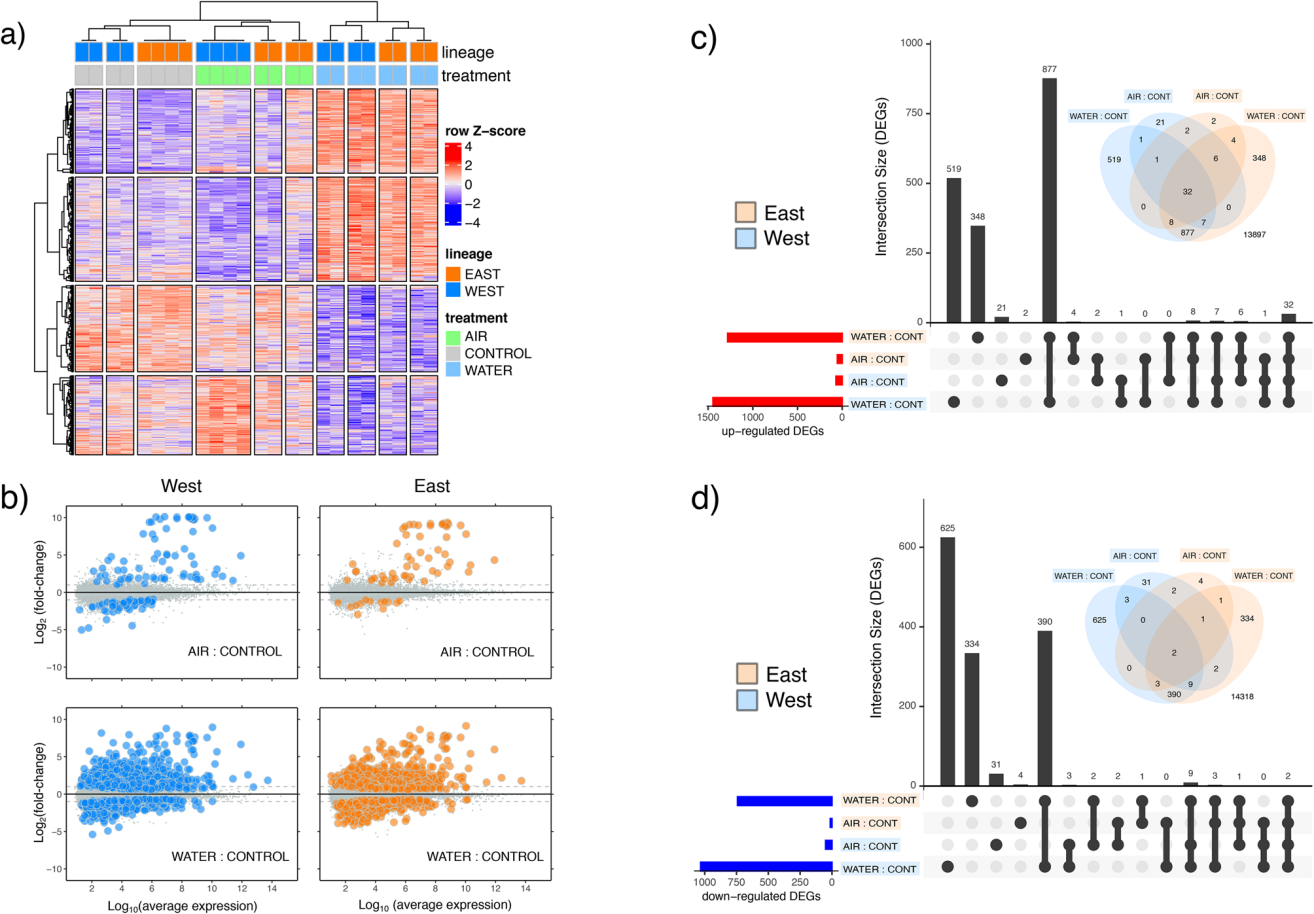
Differential gene expression. (**a**) Heatmap clustering of 4,567 DEGs differing between treatment within lineage or between lineages within a treatment (adj *P* ≤ 0.05; no fold-change cut-off). (**b**) MA plots of DEGs (coloured points; blue = West, orange = East) for AIR:CONTROL (upper plots) and WATER:CONTROL (lower plots). Intersection plots and Venn diagrams of (**c**) upregulated and (**d**) down-regulated DEGs. The intersection plots show the sizes of DEG sets for each comparison (coloured horizontal bars), and set sizes of DEGs corresponding to the Venn diagrams (vertical black bars). Heatmap was produced using R/Bioconductor package ComplexHeatMaps^42^ (http://bioconductor.org/packages/release/bioc/html/ComplexHeatmap.html).

Differential expression (DE; relative up- or down-regulation between lineages within a treatment or between treatments within a lineage) was detected for 4,567 transcripts (29% of the total transcripts analysed). Differentially expressed genes (DEGs) clustered primarily by treatment (heatmap clustering, Fig. 5a), with WATER clearly separated from AIR and CONTROL. The sizes of transcript sets responding to WATER were more than an order of magnitude larger (ca. 20-fold on average) than those responding to AIR (Fig. 5c,d). Most up-regulated DEGs in AIR also responded to high WATER temperature (64.3%), but this was reduced to 25.9% of down-regulated DEGs.

Within each treatment, there was a secondary but clear clustering by lineage. The relative effect of WATER compared to AIR exposure for both lineages is illustrated by MA plots (average expression versus fold-change; Fig. 5b). Both in terms of number of DEGs and fold-change variation, AIR and WATER exposure resulted in an excess of up-regulated over down-regulated expression relative to CONTROLs, while highly responsive DEGs (i.e., with Log_2_FC > 5) were restricted to up-regulation in response to high temperature exposure.

A set of 32 transcripts were upregulated in AIR and WATER in both lineages (Fig. 5c), forming the core of a common heat shock response (HSR; see Table S2). This core group made up a large proportion (38.1%) of the 84 transcripts upregulated in AIR in at least one lineage and 78% of the 41 transcripts upregulated in AIR in both lineages (Table 1). Further details reported in Supplementary Material.

**Table 1:** Comparative expression of molecular chaperones/co-chaperones involved in the heat stress and the unfolded protein response following exposure to AIR and WATER in Western and Eastern *P. perna* lineages.

Accession (UniProtKB)	Gene	Protein	Air:Control WEST		Air:Control EAST		Water:Control WEST		Water:Control EAST	
			log_2_(FC)	Adj *P*	log_2_(FC)	Adj *P*	log_2_(FC)	Adj *P*	log_2_(FC)	Adj *P*
A0A6J8EJ39	AHSA1	Activator of 90 kDa heat shock protein ATPase	*1.46*	*NS*	*2.43*	*NS*	**4.56**	**1.9E−05**	4.02	1.4E−04
A0A6J8B168	DNAJA1	Co-chaperone for HSPA8/Hsc70	*1.45*	*NS*	*1.97*	*NS*	2.42	3.2E−03	**2.54**	**6.8E−03**
A0A6J8AVA1	DNAJA2	Co-chaperone of Hsc70	− *0.09*	*NS*	*0.55*	*NS*	**0.99**	**2.1E−02**	0.95	3.6E−02
A0A6J8AHD5	DNAJB	Co-chaperone of the HSP70 family	**5.82**	**3.2E−04**	5.68	7.0E−04	4.28	1.2E−03	**4.86**	**5.2E−04**
A0A6J8D237	DNAJB6	Co-chaperone of the HSP70 family	*0.27*	*NS*	*0.75*	*NS*	1.05	2.5E−02	**1.17**	**1.8E−02**
A0A6J8E5G5	DNAJC7	Co-chaperone for HSP70 and HSP90	*1.10*	*NS*	*1.74*	*NS*	2.17	1.7E−03	**2.23**	**2.0E−03**
A0A1D3RM99	DNAK	Heat shock protein 70 homologue	**9.39**	**1.9E−06**	8.51	1.3E−05	6.67	5.1E−05	**6.75**	**7.2E−05**
A0A6J8EJ43	DNAK	Heat shock protein 70 homologue	*0.08*	*NS*	*0.89*	*NS*	**2.79**	**2.3E−07**	2.59	1.7E−06
A0A6J8AE36	GroEL	60 kDa heat shock protein, mitochondrial	*1.24*	*NS*	*1.81*	*NS*	**3.85**	**7.7E−07**	3.54	3.7E−06
A5A4G5	HSP70	Heat shock protein 70	**10.12**	**3.8E−06**	8.97	2.4E−05	**6.62**	**2.2E−04**	6.08	5.9E−04
B3FRR6	HSP70	Heat shock protein 70	**9.98**	**8.8E−09**	8.96	7.8E−08	7.56	2.9E−07	**7.58**	**4.8E−07**
Q4W8C7	HSP70	Heat shock protein 70	**9.99**	**7.6E−08**	9.05	4.6E−07	**7.35**	**5.4E−06**	7.23	1.1E−05
V5LVC8	HSP70	Heat shock protein 70	**9.75**	**3.3E−08**	9.17	2.1E−07	7.20	1.4E−06	**7.57**	**2.3E−06**
A0A210QAI3	HSP70B2	Heat shock protein 70	**3.75**	**9.5E−03**	3.47	2.8E−02	**5.31**	**1.4E−05**	4.47	1.7E−04
A5Y8F9	HSP71	Heat shock protein 71	*0.65*	*NS*	*0.82*	*NS*	**1.85**	**8.8E−04**	1.73	4.7E−03
C0Z203	HSP90	Heat shock protein 90	*0.88*	*NS*	*1.03*	*NS*	**2.53**	**2.0E−06**	2.17	2.8E−05
A0A6J8C161	HSPA1s	Heat shock protein 70 family	**10.07**	**2.8E−07**	9.18	9.2E−07	7.04	1.6E−05	**7.31**	**1.3E−05**
A0A6J8ERC9	HSPA1s	Heat shock protein 70 family	**9.78**	**1.3E−03**	8.97	4.3E−03	6.84	2.1E−02	**7.46**	**3.3E−03**
A0A6J8EV43	HSPA1s	Heat shock protein 70 family	**9.85**	**3.4E−08**	9.11	1.9E−07	7.62	9.4E−07	**7.77**	**1.8E−06**
A0A6J8EU81	HSPA4	Heat shock 70 kDa protein 4	4.99	2.9E−02	**5.34**	**4.8E−02**	5.54	5.7E−03	**5.57**	**1.0E−02**
A0A6J8C8Q7	HSPBP1	Hsp70-binding protein 1	*0.81*	*NS*	*3.26*	*NS*	5.20	4.1E−04	**5.50**	**2.0E−03**
F4YUB2	SHSP	Small heat shock protein	4.84	1.3E−03	**5.10**	**1.7E−03**	**6.90**	**5.1E−03**	6.36	1.6E−02
A0A6J8BJ36	SHSP domain	sHSP domain-containing protein	**6.71**	**3.0E−04**	5.90	2.0E−03	**7.71**	**4.9E−06**	7.30	1.4E−05
A0A6J8CQ51	SHSP domain	sHSP domain-containing protein	**6.55**	**7.2E−03**	6.28	1.3E−02	7.80	8.0E−06	**7.83**	**4.4E−05**
A0A6J8B610	STIP1	Stress-induced-phosphoprotein 1	*0.53*	*NS*	*1.98*	*NS*	**4.48**	**1.6E−06**	4.42	8.5E−06
A0A6J8CLN0	CCT1	T-complex protein 1 subunit alpha	*0.13*	*NS*	*1.19*	*NS*	2.18	3.0E−05	**2.47**	**4.2E−05**
K1R294	CCT2	T-complex protein 1 subunit beta	*0.39*	*NS*	*1.35*	*NS*	**2.91**	**5.8E−06**	2.84	1.6E−05
K1R466	CCT3	T-complex protein 1 subunit gamma	*0.57*	*NS*	*1.57*	*NS*	2.88	4.7E−05	**2.89**	**1.0E−04**
A0A6J8C941	CCT4	T-complex protein 1 subunit delta	*0.27*	*NS*	*1.42*	*NS*	2.49	2.7E−05	**2.68**	**2.4E−05**
A0A6J8F1K1	CCT7	T-complex protein 1 subunit eta	− *0.06*	*NS*	*0.59*	*NS*	**2.36**	**1.6E−06**	2.12	1.5E−05
A0A6J8D4A4	HSPA5	ER chaperone BiP	3.10	2.4E−02	**3.65**	**7.8E−03**	**5.93**	**2.9E−05**	5.48	4.8E−04
A0A6J8B469	DNAJC10	ER protein folding and degradation	*0.19*	*NS*	*0.82*	*NS*	1.66	3.8E−03	**1.85**	**2.5E−03**
A0A6J8D665	DNAJC3	ER Co-chaperone of HSPA8/HSC70	*0.14*	*NS*	*0.53*	*NS*	**3.13**	**3.8E−08**	2.52	1.3E−06
A0A6J8AES2	XBP1	X-box-binding protein 1	*0.09*	*NS*	*0.47*	*NS*	1.01	3.0E−03	**1.07**	**2.3E−03**
A0A6J8D3C7	ERLIN	Erlin (ERLIN1/ERLIN2 complex)	*0.00*	*NS*	− *0.09*	*NS*	**1.50**	**1.7E−03**	1.07	4.2E−02
A0A6J8A614	RNF5	E3 ubiquitin-protein ligase RNF5	− *0.11*	*NS*	*0.44*	*NS*	**1.71**	**1.6E−04**	1.16	1.8E−02
A0A6J8AHK1	PDIA4	Protein disulfide-isomerase A4	*0.44*	*NS*	*0.43*	*NS*	**3.07**	**6.6E−08**	2.57	1.3E−06
A0A6J8DZ32	ATF3	Cyclic AMP-dependent transcription factor ATF-3	*0.24*	*NS*	*1.07*	*NS*	**1.91**	**2.2E−04**	1.75	7.3E−04
A0A210PV14	EIF2AK3	Eukaryotic translation initiation factor 2-alpha kinase 3	*1.13*	*NS*	*2.25*	*NS*	**3.99**	**4.1E−05**	3.75	1.8E−04
A0A6J8BLF7	EEF2K	Eukaryotic elongation factor 2 kinase	*0.30*	*NS*	*0.51*	*NS*	**1.97**	**6.7E−05**	1.74	5.0E−04
A0A6J8F383	EIF4ENIF1	Eukaryotic translation initiation factor 4E transporter	− *0.11*	*NS*	*0.24*	*NS*	0.96	1.1E−02	**1.21**	**3.6E−03**
A0A6J8EWJ5	EIF4G	Eukaryotic initiation factor 4G family	− *0.31*	*NS*	− *0.02*	*NS*	0.96	1.9E−03	**1.06**	**7.7E−04**
A0A6J8CYA1	BNIP3L	BCL2/adenovirus E1B 19 kDa protein-interacting protein 3-like	*0.44*	*NS*	*0.06*	*NS*	**1.75**	**8.8E−05**	1.16	1.0E−02
A0A6J8DVZ2	CASP2	Caspase-2	*0.50*	*NS*	*0.43*	*NS*	**2.37**	**3.2E−06**	1.75	1.8E−04
A0A6J8EKU0	CASP7	Caspase-7	*0.05*	*NS*	*0.94*	*NS*	1.67	1.2E−02	**1.79**	**1.0E−02**
A0A385L2T9	TRAF3	TNF receptor-associated factor 3	*0.07*	*NS*	− *0.83*	*NS*	**1.75**	**1.9E−02**	0.98	9.5E−03
A0A6J8C5R0	TNFAIP3	Tumor necrosis factor alpha-induced protein 3	− *0.49*	*NS*	− *0.04*	*NS*	**1.66**	**3.5E−05**	1.47	4.0E−04
A0A6J8DF49	DNAJA3	Modulates apoptotic signal transduction in mitochondria	*0.01*	*NS*	*0.41*	*NS*	1.11	1.6E−03	**1.40**	**3.2E−04**
K1QQZ1	DNAJA3	Modulates apoptotic signal transduction in mitochondria	*0.13*	*NS*	*0.07*	*NS*	**1.24**	**4.7E−03**	1.04	4.2E−02
A0A6J8DIZ2	PGRP	Peptidoglycan recognition protein 1	*0.39*	*NS*	− *0.26*	*NS*	**1.51**	**7.1E−03**	1.49	9.5E−03

All accessions were significantly upregulated in response to WATER (not AIR when indicated by NS). The greatest fold-change values (log2, bold) by lineage within each treatment are indicated, together with BH-adjusted *P *values.

Significant values are in italics.

#### Expression breadth is greater in the Western lineage

Set sizes of DE transcripts (Fig. 5c,d: horizontal coloured bars) were larger in response to both thermal stressors in the Western than the Eastern lineage. By treating Fold Change (FC) between treatments and controls as a continuous variable (“expression breadth”) and using sets of significantly DE transcripts in AIR or WATER in either or both lineages, we asked whether FC distributions of up- or down-regulated DEGs differed between Western and Eastern lineages. Two-tailed paired T-tests for FC values between lineages revealed a significantly higher absolute FC response for the Western lineage under both AIR and WATER treatments relative to control conditions (Fig. S4).

#### GO enrichment analysis

**Figure 6:**
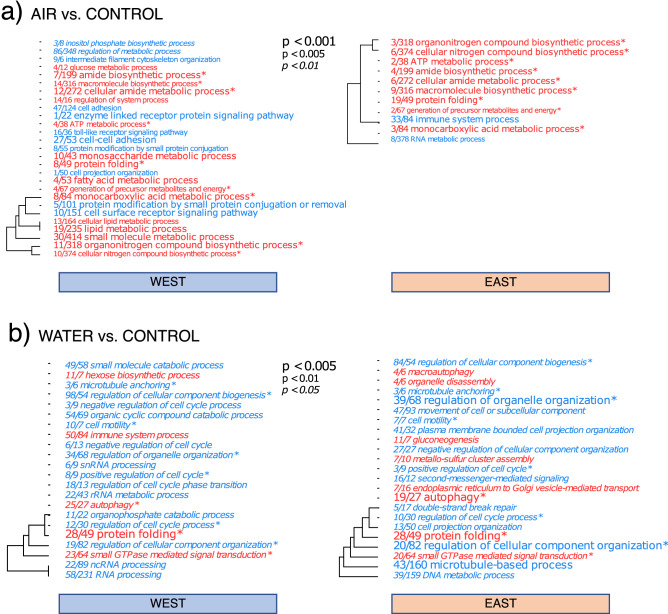
Gene ontology (GO) enrichment analysis in the Biological process category in response to (**a**) AIR and (**b**) WATER versus the respective controls for the Western and Eastern lineages (left and right panels, respectively). Up-regulated GO terms are shown in red and down-regulated in blue. Size and style of the text corresponds to the significance level (Mann–Whitney U-tests on ranked genes) shown in the legends. Hierarchical clustering trees indicate gene sharing between GO categories; zero-length branches are subsets of each other. The fractions preceding GO terms indicate the numbers of annotated genes passing/failing an unadjusted *P* value threshold of 0.05. Asterisks (*) depicts common GO terms between lineages.

A considerable number of GO terms were enriched in AIR vs. CONTROL responses, especially given the relatively low level of transcript-specific differential expression (Fig. 6a, *cf* Fig. 5b–d). Several related terms were shared by both lineages, including down-regulation of innate immune responses and toll-like receptor signalling, upregulation of protein folding, as well as some general terms involving organic nitrogen metabolism (Fig. 6a, Fig. S5). However, the Western lineage also included terms absent in the Eastern lineage, e.g., those related to lipid and fatty acid metabolism (upregulated) and cell–cell adhesion (down-regulated).

In WATER vs. CONTROL comparisons, GO terms for cytoskeletal functions and cell cycle processes were down-regulated, while protein folding and autophagy were upregulated, together with the generation of sugars via gluconeogenesis (“hexose biosynthetic process” [West] and “gluconeogenesis” [East]; Fig. 6b). In the Western lineage (and in contrast to AIR exposure) there was also an indication of immune system upregulation, and a somewhat stronger upregulation of genes involved in autophagy (Fig. 6b). In general, where common GO terms were found in both lineages for either stressor, more significant term members (≈ genes) were detected in the Western compared to the Eastern lineage suggesting greater expression breadth in the former (*cf.* Fig. 6 and Fig. S3).

#### Weighted gene correlation network analysis co-expression modules associated with lineage-specific differences

The 5995 DEGs (unadjusted *P* < 0.1) used to construct a co-expression network identified 8 modules. Two of these, the “blue” (containing the majority of the genes; 5069) and “brown” modules (190 genes) were positively and negatively correlated, respectively, with samples exposed to WATER in a largely lineage-independent manner and were not analysed further. Of the remaining 6 modules, 5 were strongly correlated with a treatment in one of the 2 lineages (Fig. S6). Module eigengenes (ME) of modules, which may contain both up- and down-regulated genes, were correlated with traits (treatment conditions). Significant eigengene module-trait correlations may be positive or negative, depending on whether member genes show expression patterns in the same or opposite direction in the module and trait samples.

The eigengene of the largest module (“greenyellow”, 329 genes) was positively correlated with AIR exposure in the Western lineage (Pearson’s *R*^2^ = 0.72; *P* = 6e−5, Fig. S7a). The “black” module (97 genes) was negatively correlated with the same samples (Pearson’s *R*^2^ = -0.81; *P* = 2e−6), and to a much lesser extent with AIR in the Eastern lineage (Fig. S7b). Two other modules, “midnightblue” (130 genes, Pearson’s *R*^2^ = -0.66; *P* = 5e−4) and “grey60” (36 genes, Pearson’s *R*^2^ = -0.77; *P* = 1e−5) were negatively correlated with WATER in the Western and Eastern lineages, respectively (Fig. S7c,d). Finally, the “cyan” module (51 genes) was negatively correlated with CONTROL samples in the Western lineage (Pearson’s *R*^2^ = -0.83; *P* = 7e−7; Fig. S7e). Gene enrichment analysis (GO-MWU) was unsuccessful in detecting significantly enriched terms for WGCNA modules, likely due to their modest size.

**Figure 7:**
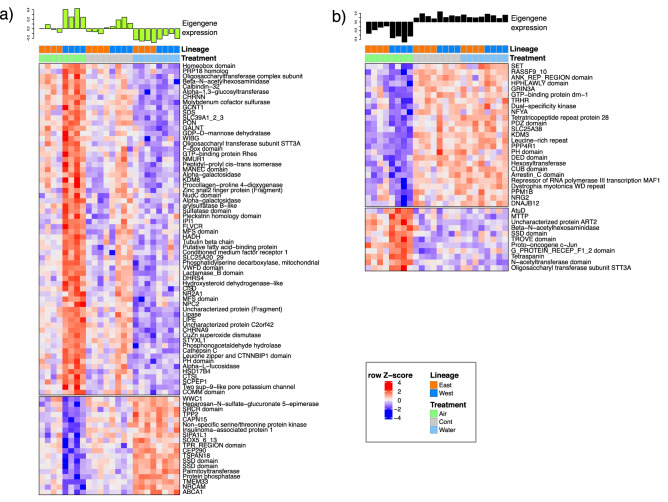
Gene expression heatmaps of annotated transcripts (MM > 0.6, GS > 0.6) in (**a**) green yellow and (**b**) black modules. The per sample eigengene expression is shown above each heatmap showing the correlation with AIR samples from the western lineage. Heatmap colours are scaled by row Z-score; blue = downregulated, red = upregulated genes. Coloured bars at the head of each heatmap indicate the experimental treatment and lineage for each sample as indicated in the legend. Heatmap was produced using R/Bioconductor package ComplexHeatMaps^42^ (http://bioconductor.org/packages/release/bioc/html/ComplexHeatmap.html).

Genes with both high module membership and gene significance for the trait (defined as MM and GS > 0.6, Fig. S7) were selected and used to construct heatmaps of sample gene expression with respect to module eigengene expression. Expression changes in both the “greenyellow” and “black” modules in the Western lineage in response to AIR are either unresponsive or much less so in the Eastern lineage (Fig. 7a,b). Furthermore, they tend to be regulated in the opposite way in response to WATER, indicating that they are specifically activated/repressed and do not form part of a species-wide or general thermal response. In these modules we found several upregulated genes involved in signal reception and transduction or transport across membranes, including G protein-coupled receptors, ion channels (neurotransmission and K^+^), heme, and Zn.

An acylcarnitine transporter, together with genes for fatty acid beta oxidation were upregulated, as were genes for oxidative stress (Cu/Zn superoxide dismutase), inflammatory response and apoptotic signaling pathways. The increased expression of various genes involved in glycosaminoglycan (GAG) synthesis and degradation suggests that remodelling of GAGs is particularly prominent in the western lineage in response to aerial exposure, while cell division, differentiation and proliferation-related genes were among those down-regulated.

**Figure 8:**
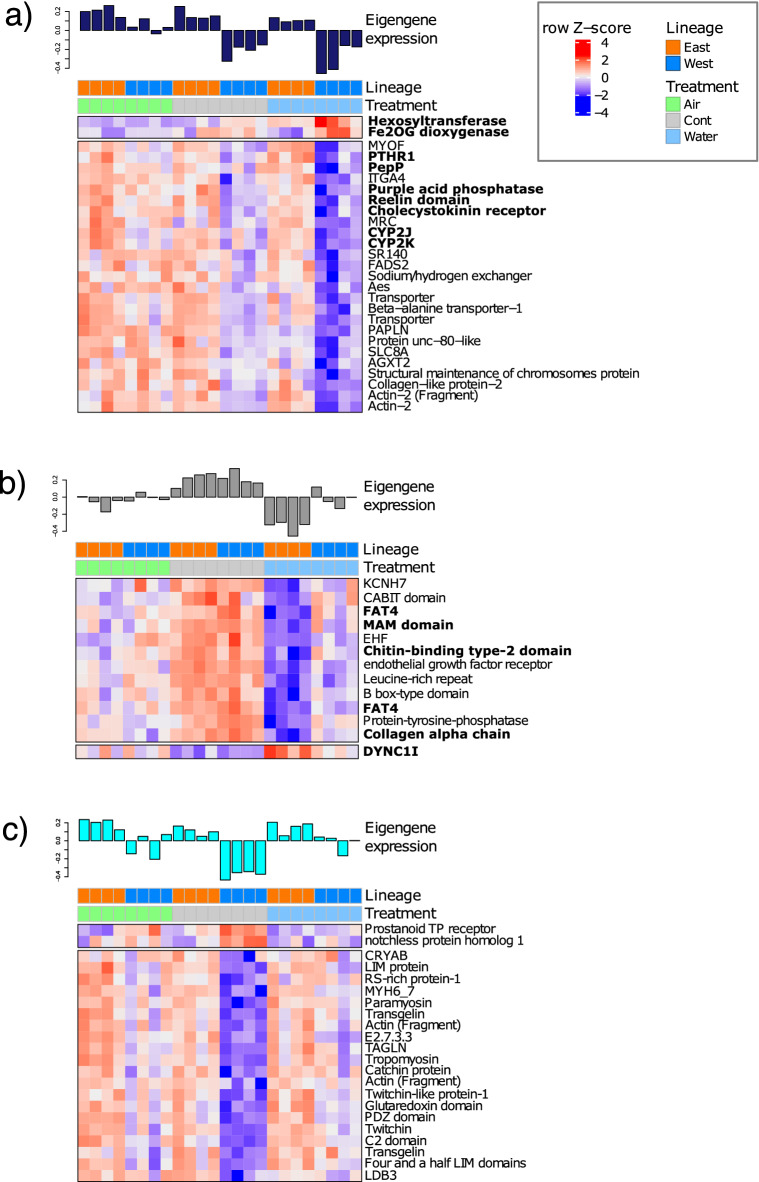
Gene expression heatmaps of annotated transcripts (MM > 0.6, GS > 0.6) in three WGCNA modules. The per sample eigengene expression is shown above each heatmap showing the correlation with WATER samples from (**a**) the western and (**b**) eastern lineages, and with (**c**) CONTROL from the western lineage. Heatmap colours are scaled by row Z-score; blue = downregulated, red = upregulated genes. Genes shown in bold are discussed in the text. Coloured bars at the head of each heatmap indicate the experimental treatment and lineage for each sample as indicated in the legend. Heatmap was produced using R/Bioconductor package ComplexHeatMaps^42^ (http://bioconductor.org/packages/release/bioc/html/ComplexHeatmap.html).

Two modules showed significant correlations with WATER in the Western (“midnightblue”) and Eastern lineages (“grey60”), respectively. The majority of genes belonging to these modules were downregulated in response to WATER (Fig. 8a,b): in the case of the Western lineage, there was a weak lineage-specific association of the module eigengene with CONTROL samples (Fig. 8a, Figs S6, S7d), which was absent in the case of the Eastern lineage (Fig. 8b, Figs. S6, S7d). The Western lineage was notable for the downregulation of several genes involved in nervous system development and function (PepP, Reelin domain, Cholecystokinin receptor), Ca^2+^ regulation (PTHR1), as well as 2 cytochrome 450 family members (CYP2J, CYP2K) and lysosomal acid phosphatase (Purple acid phosphatase). The two upregulated genes were involved in oxygen-sensing and protein glycosyation (Fig. 8a). By contrast, downregulated Eastern lineage genes were mainly associated with cell adhesion and the extracellular matrix (FAT4 cadherins, MAM domain, collagen and chitin-binding protein), in addition to transcription factors. A dynein heavy chain (intracellular transport) was the only upregulated gene.

A small set of genes represented by the “cyan” module was markedly associated with the Western lineage CONTROL samples (Fig. 8c, Figs. S6, S7e). A prostanoid receptor with potential roles in cardiovascular/immune system and possibly sensory perception, and notchless protein homolog 1 (a member of the Notch signaling pathway) were upregulated, while several genes with muscle contractile functions (Tropomyosin, Paramyosin, Twitchin) or cytoskeletal associations were downregulated.

## Discussion

We combined measures of phenotypic plasticity (survival curves) with molecular phenotyping (gene expression profiling) to highlight three key results. First, the two genetic lineages have clearly different thermal tolerances reflecting their current distributions and thermal niches. Second, transcriptional profiles showed similar patterns of expression indicating a baseline, common response to thermal stress at the species level, with a stronger response in water than in air. Third, and most importantly, we identified lineage-specific differences in the underlying transcriptional pathways in response to warmer temperatures, that potentially link to differences in thermal tolerance. Lastly, a phylogenomic analysis of 407 single copy protein coding loci confirmed the recent phylogeographic break between eastern and western phylogroups identified by Cunha et al.^20^.

### Different thermal tolerances between genetic lineages

After acute high temperature stress, the Eastern lineage showed much higher survival than the Western. During aerial exposure in the low tide simulation at 39 °C, fifty percent of Western individuals had died by the end of the experiment, after 84 h, while similar rates of mortality were reached only at 41 °C by the Eastern lineage. A similar, stronger pattern was observed during heat stress in water. At 27 °C, the Western lineage suffered fifty percent mortality after 141 h, while the Eastern showed zero mortality even after 186 h.

These findings support previous studies^7,21^, demonstrating that the Eastern lineage is better adapted to warm water conditions. Indeed, the Western lineage occurs on the Namibian coast and the warm-temperate south coast of South Africa^52^, while the eastern lineage occurs on the subtropical southeast and east coasts of South Africa. Moderate gene flow between the lineages has been detected between the subtropical and warm-temperate bioregions and, critically, this flow is predominantly east-to-west (i.e., warmer to cooler; Zardi et al.^2^). Our results indicate that such directional gene flow can be explained by local selection on the east coast where the low thermal tolerance of the Western lineage limits genetic admixture on the subtropical coast.

A second, non-exclusive explanation for the direction of gene flow is that oceanographic features make larval dispersal directional. The Agulhas Current dominates nearshore waters of the east and south coasts of South Africa^53^. At the transition between the temperate and subtropical regions, the inshore thermal front of this powerful southwest-flowing current (10–20 km day^−1^) usually lies very near to the shore 0–1 km offshore^54^, consequently influencing the along-shore transport of larvae in this area^55^. Evidence from oceanographic drifters^21^ and Lagrangian Particle Simulations^56^ indicates that that most larvae approaching the region of the genetic break are either advected offshore by the Agulhas Current and lost, or transported east-to-west.

### Thermal stress induced a greater transcriptional response in water than in air

Mussels exposed to thermal stress in air and seawater showed an excess of up-regulated over down-regulated expression relative to control individuals. Exposure to high temperatures triggered a core heat shock response common to both lineages. In particular, the over expression of transcripts for HSP70, sHSP and DnaJ supports research highlighting the pivotal role of heat shock proteins (Hsp) as molecular chaperones that mitigate both internal and external stress^57–59^.

Importantly, the transcriptional response was stronger in water than air, with transcript sets that were significantly larger (> 1 order of magnitude) and a broader range of functional responses that involved cellular homeostasis and metabolic regulation, signal perception and transduction, protein and DNA damage, apoptosis and autophagy.

Under heat stress in seawater, mussels showed a more pronounced unfolded protein response (UPR) than in air. UPR is an intracellular signalling pathway initiated by the accumulation of unfolded proteins in the endoplasmic reticulum ER^60^. UPR constitutes a central intracellular control mechanism that adjusts organelle abundance in response to environmental clues by triggering a wide-ranging transcriptional response that regulates the ER protein folding capacity. Together, these results suggest either a more acute thermal stress response in seawater than in air, and/or chronic effects prolonging upregulation during the recovery period following the stress.

Previous studies have shown that major biogeographic patterns of intertidal species are especially susceptible to increases in water temperature while elevated air temperature plays a secondary role e.g.,^10,61^. This may be due to evaporative cooling of body temperatures, and thus stress, in air^62,63^. Given the distinctive natures of air and water as media, tissue heating and cooling rates differ significantly between the two treatments, and, indeed, in nature. Abundant evidence from high frequency in situ measurements using biomimetic sensors, Infrared imagery and digital thermocouples shows that mussel body temperatures increase more gradually during low tide aerial exposure than during immersion at high tide^21,64,65^.

Behaviour can also mitigate heat stress in air. Intertidal habitats exhibit extreme spatial and temporal variability and mussels display a variety of behaviours that can minimise stress e.g.,^66,67^. *P. perna* gapes the shell with alternate closure and opening of the shell during emersion^68^. These valve movements push water out of the mantle cavity, increasing the risk of desiccation, but also significantly reducing body temperatures of the individual and whole aggregations of mussels through evaporative cooling^28^.

Importantly, the temperatures used for the air and water thermal stress treatments, 40 °C and 30 °C respectively, are representative of maximum sublethal temperatures where the distributional ranges of the two *P. perna* two lineages overlap^21,26^. However, it is critical to note that that different water and air temperatures used in our study may also contribute to the relatively lower stress transcriptional response observed during thermal stress in air.

### Lineage-specific differences in the transcriptional response to thermal stress

We detected expression changes in the Western lineage in response to aerial thermal stress that were either absent or much weaker in the Eastern lineage. Specifically, genes related to signal reception and transduction or transport across membranes as well as genes linked to oxidative stress, inflammatory responses and apoptotic signalling pathways were upregulated. Abundant literature demonstrates that heat stress can initiate multiple deleterious physiological effects, such as endocrine disorders^69,70^, electrolyte imbalance^71^, immune dysfunction^72,73^, and oxidative stress^74,75^. The pathway leading to such heat stress responses is partly triggered by elevated levels of pro-inflammatory cytokines and reactive oxygen species ROS^76^. Higher production of ROS stimulates intracellular and extracellular superoxide formation and is ultimately responsible for oxidative stress^77^. Heat stress-induced overproduction of ROS can modulate the inflammatory transcription factor NF-κB^78^, causing hormonal and metabolic changes and decreasing the level of antioxidant enzymes^79^. Further, previous studies have shown that higher levels of both pro-inflammatory cytokines and ROS are inflammatory mediators^80^. Critically, exposure to heat causes mitochondrial dysfunction^81,82^. Under normal conditions, damaged mitochondria would be removed through selective autophagy of mitochondria, known as mitophagy^83^. Mitophagy is of central importance to maintaining mitochondrial function in order to prevent production of excess ROS, and ultimately promotes cell survival under heat stress. Inhibition of or reductions in mitophagy produce increasing levels of ROS, leading to free radical injury, and apoptotic signalling^84,85^.

In contrast to upregulated genes, cell division-, differentiation- and proliferation-related genes were down-regulated, though again especially in the Western lineage. This transcriptional remodelling suggests that an energy deficit could occur, with programmed cell death (apoptosis) triggered when cellular viability and membrane integrity are compromised by heat stress.

The stronger stress response in the Western lineage could be a sign of either higher levels of cell damage or a relatively high capacity of the lineage for dealing with such damage. Theoretically, the capacity to initiate a stronger stress response, and to do so rapidly, has been suggested as a mechanistic basis for greater tolerance in stress-resistant species^86^, but our survival experiments confirmed the lower tolerance of the Western lineage. Previous studies have also shown that differences in behavioural traits help explain the exclusion of Western individuals from the east coast while oceanographic barriers explain the exclusion of the Eastern lineage from the south-west coast^7,21^. Taken together, this suggests that the downstream consequences of early cell-signalling events may play important roles in limiting an eastward expansion of the Western lineage and maintaining its current distributional range.

When exposed to thermal stress in seawater, both lineages showed downregulation of a set of genes with, however, substantial differences. In Western mussels, several genes involved in nervous system development and function, Ca^2+^ regulation, as well as 2 cytochrome 450 family members and lysosomal acid phosphatase were downregulated. Heat stress can strongly affect all biological systems, and evidence from several taxa suggests that the nervous system is more sensitive to heat than other body tissues e.g.,^87,88^. At the cellular level, heat can directly produce nervous tissue injury with significant behavioural and physiological consequences^89^. Downregulation of genes implicated in the development and function of the nervous system has been reported in response to various forms of stress, including oxidative and heat stress^90^ and it has been suggested that the induction of reduced gene expression by heat is associated with neuronal cell death^91^.

Gene downregulation in Eastern mussels was, however, mainly associated with transmembrane adhesion molecules and the extracellular matrix, which mediate cell–cell adhesion and thus play an essential role in tissue integrity^92^. Failure to form adequate cell–cell adhesion contacts predisposes cells to proteasome inhibition-induced cell death^92^. Critically, heat shock transcription factors are key regulators of transmembrane adhesion molecules that mediate Ca^2+^-dependent cell–cell adhesion, such as cadherins^92^ and it has been demonstrated that lack of heat shock transcription factors can lead to a profound downregulation of cadherins at both the mRNA and protein levels^92^.

## Conclusion

Under climate change, extreme heat events will increasingly threaten the planet’s biodiversity. We show that, under heat stress, particularly in water, both survival and transcriptomic responses differed between two lineages of an abundant intertidal mussel. In this context, within-species variation is an important component of biodiversity as it may include diversity in sensitivity to and resilience of heat stress among conspecific individuals or populations. Understanding how mechanistic and cellular responses to thermal stress vary within-species and identifying genes potentially involved in responses to temperature stress allows the identification of distinct, evolutionarily divergent units in conservation actions and better predictions of the consequences of climate change for the species as whole.

## Supplementary Information

Supplementary Figure S1.

Supplementary Figure S2.

Supplementary Figure S3.

Supplementary Figure S4.

Supplementary Figure S5.

Supplementary Figure S6.

Supplementary Figure S7.

Supplementary Information 8.

Supplementary Table S1.

Supplementary Table S2.

Supplementary Table S3.

## Data Availability

The data that support the findings of this study are openly available in Dryad at https://datadryad.org/stash/share/kZLDmWjOIQ-kD9gnUW4B3PoEHwtpnS7rPbSazyq8MZU, and in NCBI's BioProject database, reference number PRJNA795292.
