# Supplementary figures and images for: Transcriptome wide analyses reveal intraspecific diversity in thermal stress responses of a dominant habitat‐forming species
Source: Sci Rep. 2023 Apr 6;13:5645. doi: 10.1038/s41598-023-32654-w (PMC10079687; doi:10.1038/s41598-023-32654-w)

Figure S1.

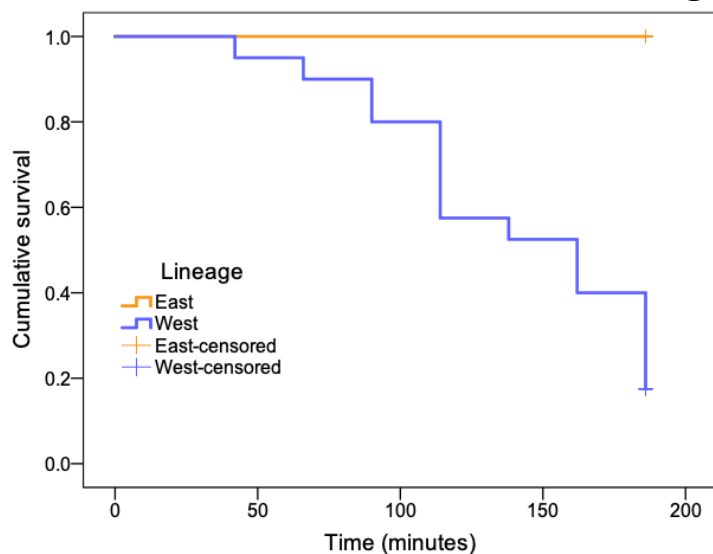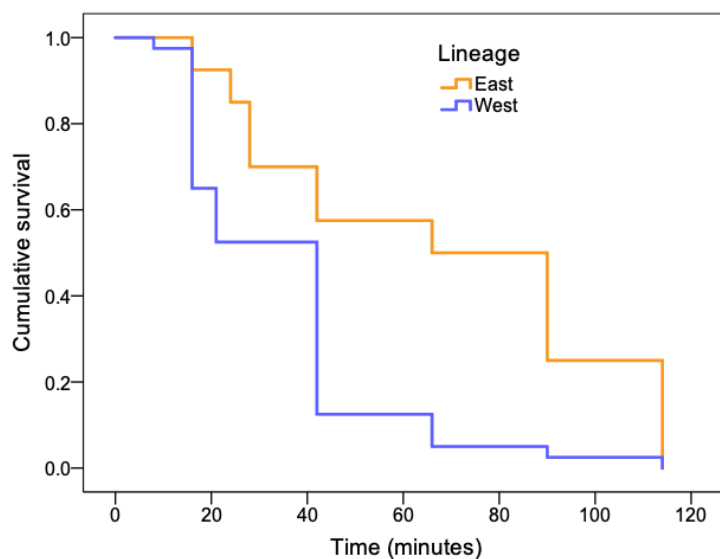

Figure S1. The survival probabilities for each lineage when in seawater at (A) 27°C and (B) 31°C

Supplement: Supplementary file 1 — Supplementary Figure S1. [file 41598_2023_32654_MOESM1_ESM.pdf]

Figure S2.

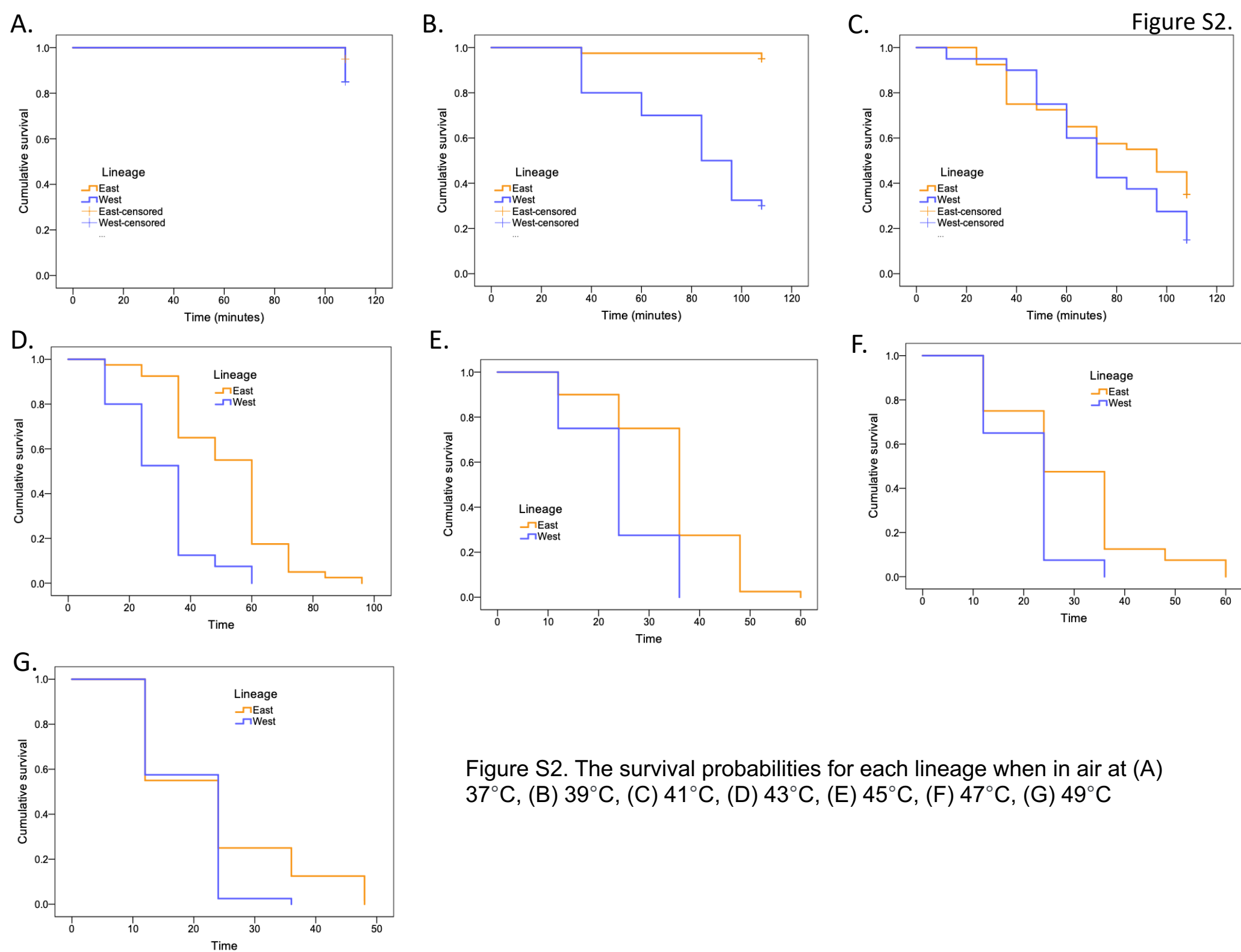

Supplement: Supplementary file 2 — Supplementary Figure S2. [file 41598_2023_32654_MOESM2_ESM.pdf]
