# Supplementary material for: Transcriptome wide analyses reveal intraspecific diversity in thermal stress responses of a dominant habitat‐forming species
Source: Sci Rep. 2023 Apr 6;13:5645. doi: 10.1038/s41598-023-32654-w (PMC10079687; doi:10.1038/s41598-023-32654-w)
Supplement: Supplementary file 3 — Supplementary Figure S3. [file 41598_2023_32654_MOESM3_ESM.pdf]

Figure S3

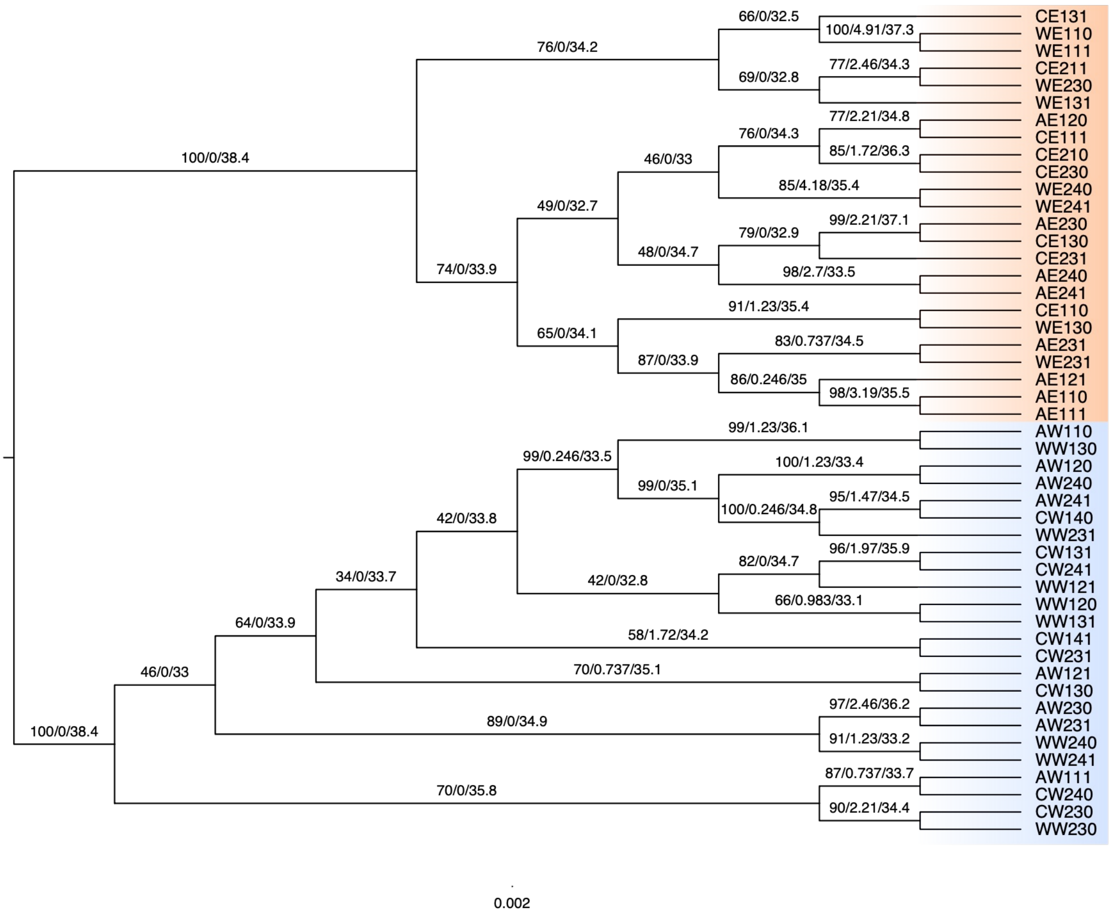

Figure S3. Cladogram representation of the maximum likelihood edge-linked partition analysis for the sample alleles used in this experiment, with concordance factors for genes (gCF) and sites (sCF). The tree was built from a concatenated supermatrix of aligned protein coding regions (434,025 sites) derived from 407 BUSCO single-copy orthologues. Branch values show bootstrap support/gCF/sCF values.
