# Supplementary material for: Transcriptome wide analyses reveal intraspecific diversity in thermal stress responses of a dominant habitat‐forming species
Source: Sci Rep. 2023 Apr 6;13:5645. doi: 10.1038/s41598-023-32654-w (PMC10079687; doi:10.1038/s41598-023-32654-w)
Supplement: Supplementary file 4 — Supplementary Figure S4. [file 41598_2023_32654_MOESM4_ESM.pdf]

Figure S4

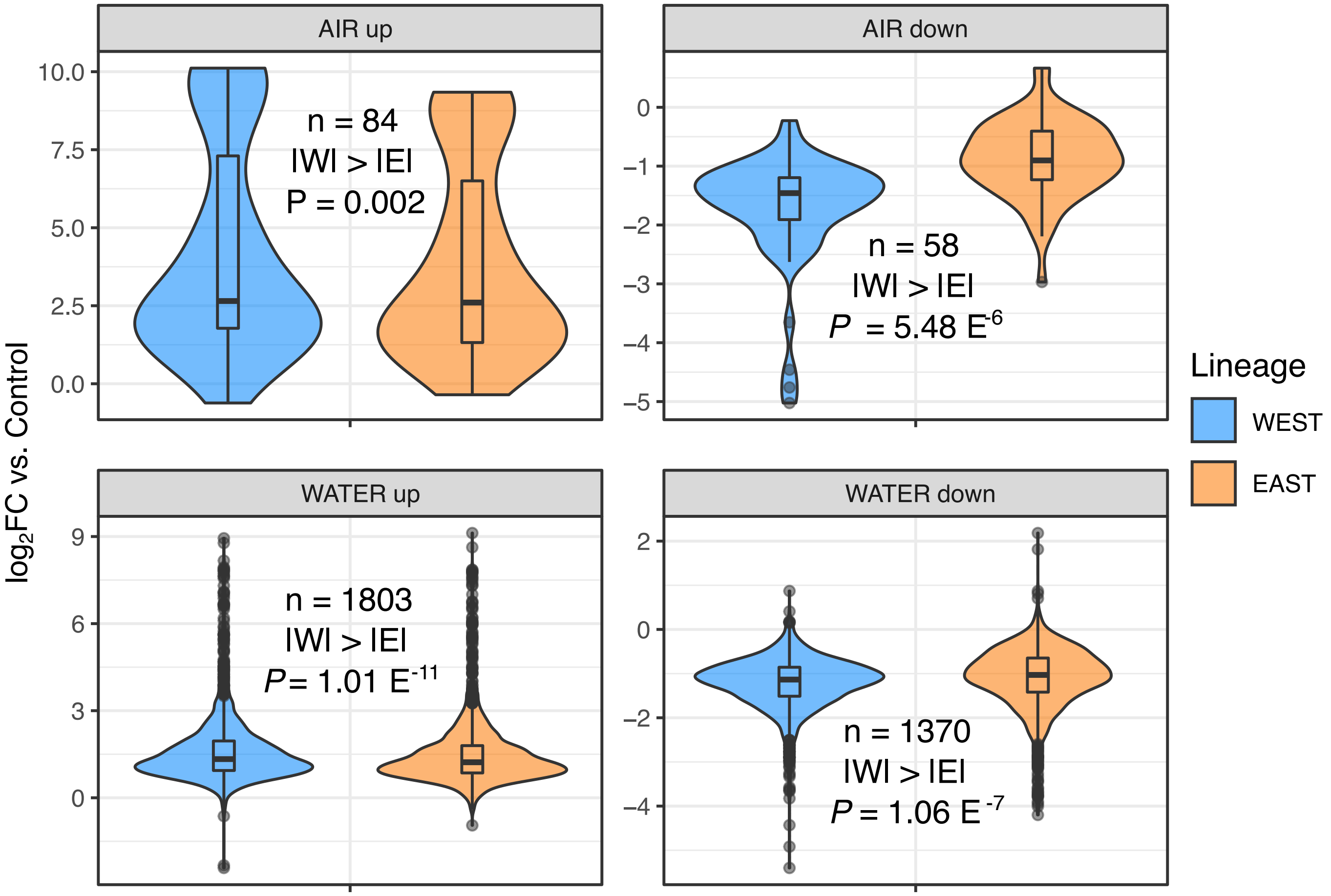

Figure S4. Fold change (FC) distributions for sets of DEGs in AIR and WATER. The panels show up- (left panels) and down-regulation (right panels) in response to AIR (upper panels) and WATER (lower panels) of common sets of DEGs in either/both the Western and Eastern lineages. The results of paired T-tests (2-tailed) between lineages show that absolute FC (|FC|) is higher in the Western lineage (|W|) than in the Eastern lineage (|E|) in each of the 4 transcript sets.
