# Supplementary material for: Transcriptome wide analyses reveal intraspecific diversity in thermal stress responses of a dominant habitat‐forming species
Source: Sci Rep. 2023 Apr 6;13:5645. doi: 10.1038/s41598-023-32654-w (PMC10079687; doi:10.1038/s41598-023-32654-w)
Supplement: Supplementary file 5 — Supplementary Figure S5. [file 41598_2023_32654_MOESM5_ESM.pdf]

Figure S5

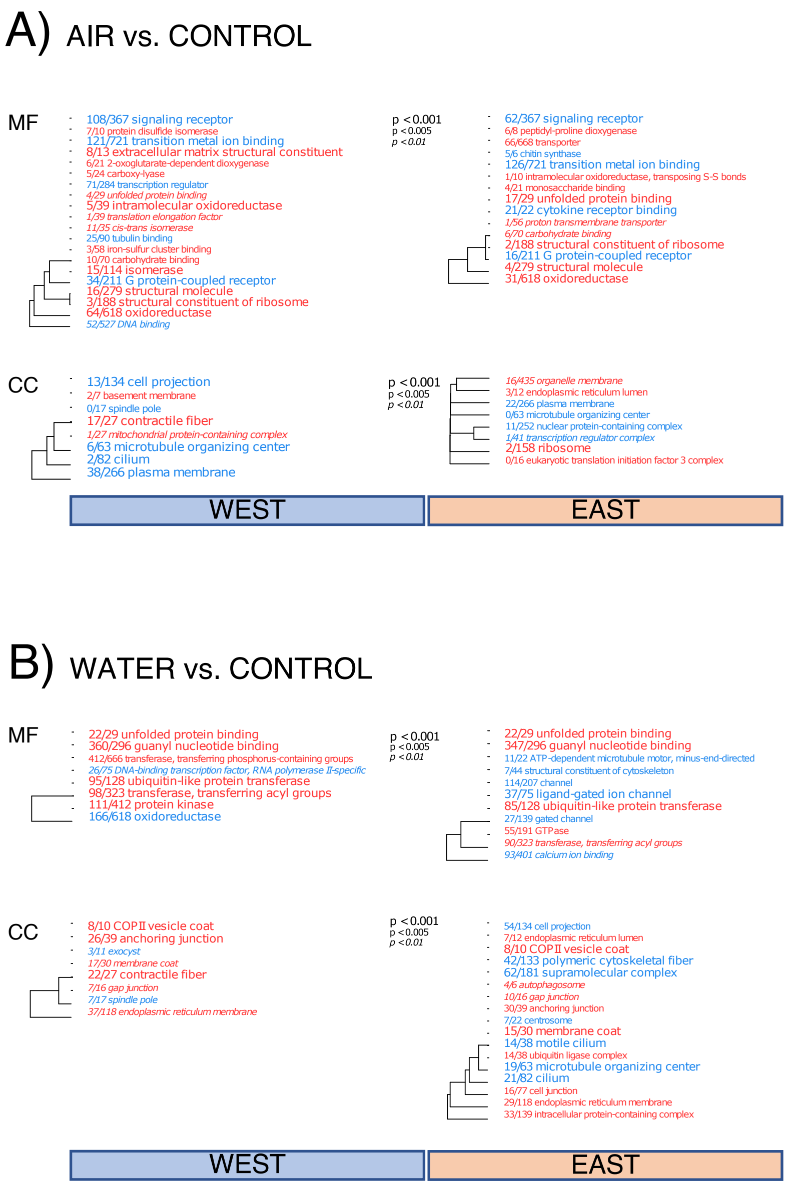

Figure S5. Gene ontology (GO) enrichment analysis in the Molecular function and Cellular compartment categories in response to A) AIR and B) WATER versus the respective controls for the Western and Eastern lineages (left and right panels, respectively). Up-regulated GO terms are shown in red and down-regulated in blue. Size and style of the text corresponds to the significance level (Mann-Whitney U-tests on ranked genes) shown in the legends. Hierarchical clustering trees indicate gene sharing between GO categories; zero-length branches are subsets of each other. The fractions preceding GO terms indicate the numbers of annotated genes passing/failing an unadjusted *P*-value threshold of 0.05.
