# Supplementary material for: Transcriptome wide analyses reveal intraspecific diversity in thermal stress responses of a dominant habitat‐forming species
Source: Sci Rep. 2023 Apr 6;13:5645. doi: 10.1038/s41598-023-32654-w (PMC10079687; doi:10.1038/s41598-023-32654-w)
Supplement: Supplementary file 6 — Supplementary Figure S6. [file 41598_2023_32654_MOESM6_ESM.pdf]

Figure S6

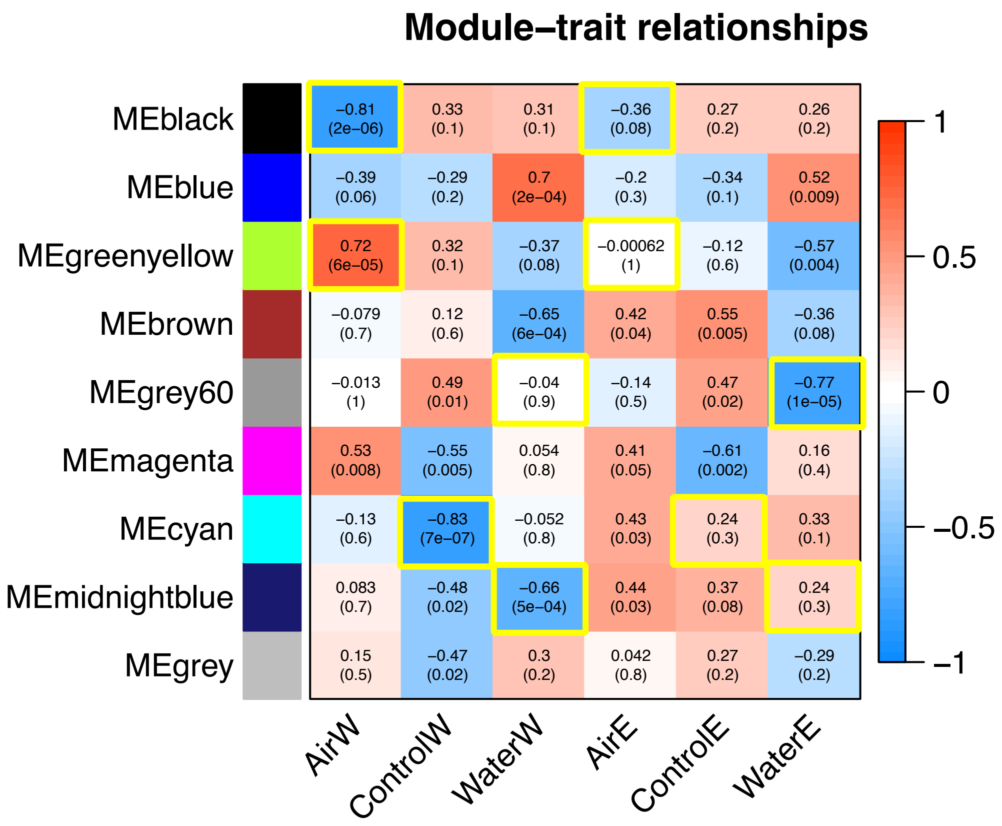

Figure S6. Module-trait correlation coefficients (Pearson's R) and *P*-values in parentheses from WGCNA analysis. W = Western and E = Eastern lineage. Yellow borders highlight modules with lineage-specific variation.
