# Supplementary material for: Transcriptome wide analyses reveal intraspecific diversity in thermal stress responses of a dominant habitat‐forming species
Source: Sci Rep. 2023 Apr 6;13:5645. doi: 10.1038/s41598-023-32654-w (PMC10079687; doi:10.1038/s41598-023-32654-w)
Supplement: Supplementary file 7 — Supplementary Figure S7. [file 41598_2023_32654_MOESM7_ESM.pdf]

Figure S7

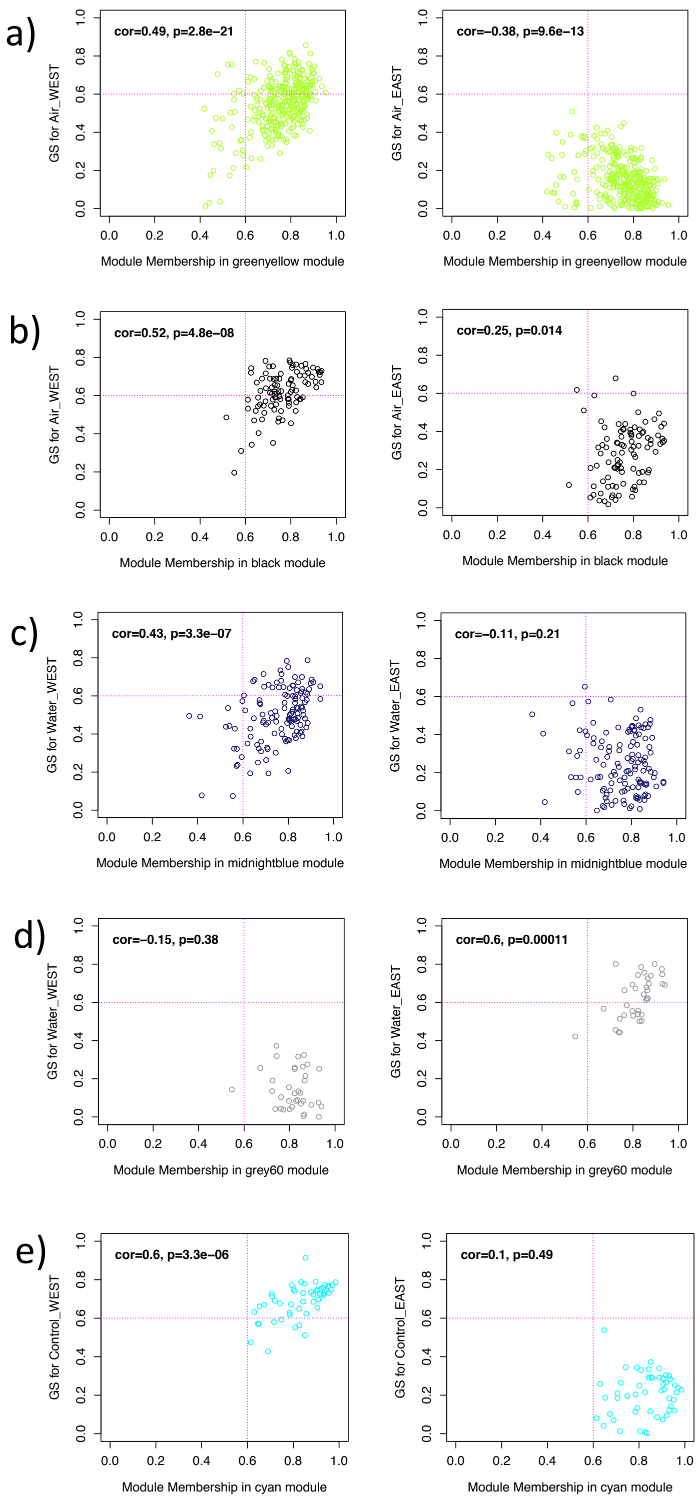

Figure S7. Relationships between individual genes' module membership (x-axis; correlation with the module eigengene) and significance for the disease state (y-axis; correlation of the gene with the treatment by lineage, WEST to the left and EAST to the right). Open circles on plots are values for individual genes. The upper right quadrant on the plots delimits the region encompassing genes with both module membership and gene significance scores higher than 0.6. Pearson correlation values and the p-value of the correlation test are indicated in the upper-left of each scatterplot.
