# Supplementary material for: Transcriptome wide analyses reveal intraspecific diversity in thermal stress responses of a dominant habitat‐forming species
Source: Sci Rep. 2023 Apr 6;13:5645. doi: 10.1038/s41598-023-32654-w (PMC10079687; doi:10.1038/s41598-023-32654-w)
Supplement: Supplementary file 8 — Supplementary Information 8. [file 41598_2023_32654_MOESM8_ESM.docx]

**Supplementary Material**

***RESULTS***

***Transcript-level expression of thermal stress responses in air vs. water***

A set 32 transcripts were upregulated in AIR and WATER in both lineages (Fig. 5c), forming the core of a common heat shock response (HSR; see Table S2). This core group made up a large proportion (38.1%) of the 84 transcripts upregulated in AIR in at least one lineage and 78% of the 41 transcripts upregulated in AIR in both lineages.

Annotated members included multiple transcripts for HSP70 (7 accessions), sHSP (3 accessions) and DnaJ. Consistent with a core HSR, transcripts encoding the co-chaperone BAG3, which plays an important regulatory role in cellular responses to several stressors, including the nuclear translocation of heat shock transcription factor 1 (HSF1) and subsequent expression of heat shock proteins (Jin et al. 2015) were upregulated. Other upregulated transcripts included sequestosome-1 (SQSTM1), a receptor required for macroautophagy and an activator of the NFE2L2/NRF2 pathway involved in cell responses to oxidative stress and expression of cytoprotective genes. The core group also contained transcripts annotated as basic leucine zipper (BZIP) and Krueppel-like factor 5 (KLF5, GC-box binding) transcription factors (UniProt genes MCOR_44127 and MCOR_17717, respectively, from *Mytilus coruscus*).

Consistent with broader and more extensive DE, thermal stress in WATER induced a wider range of functional responses compared with AIR. Pathways and processes ranged from signal perception and transduction, metabolic regulation and homeostasis, to cellular stress responses, inhibition of protein synthesis, protein degradation and DNA damage, immune and inflammatory responses, apoptosis and autophagy (Table S3). Despite fold-changes for several chaperones being even higher in AIR than WATER (e.g., several HSP70s, Table 1), following thermal stress in WATER, genes involved in endoplasmic reticulum (ER) stress and the unfolded protein response (UPR) were uniquely upregulated (Table 1). The UPR is triggered when unfolded/misfolded proteins accumulate in the ER. In mammalian systems, signals from the ER trigger several pathways, including increased transcription of ER chaperones, ER-associated degradation (ERAD), autophagy/apoptosis, as well as the global repression of protein synthesis and the cell cycle. The key ER chaperone BiP (HSPA5) was upregulated ca, 9-12-fold in AIR, but 45-60-fold in WATER, while DNAJ family ER chaperones/co-chaperones were significantly upregulated only in WATER. The following genes were also upregulated specifically in response to WATER: 1) The key UPR transcriptional regulator, X-box-binding protein 1 (XBP1), which initiates specific transcription of ER chaperones, ERAD components (e.g., ERLIN, RNF6) and inflammatory response genes (Cao & Kaufman, 2012) (Table 1). 2) Cyclic AMP-dependent transcription fact ATF3, 3) Eukaryotic translation initiation factor 2-alpha kinase 3 (EIF2AK3, PERK homologue), which is induced by ER stress and dimerizes to release BiP and phosphorylates eIF-2-alpha, promoting the global inhibition of protein synthesis. 4) Other upregulated regulators of protein synthesis include Elongation factor 2 kinase (EEF2K), Eukaryotic translation initiation factor 4E transporter (EIF4ENIF1), both negative regulators. and Eukaryotic initiation factor 4G (EIF4G), which is required for ER stress-induced ATF4 transcription factor translation.

This suggests either a more acute thermal stress response in WATER and/or chronic effects leading to an extended period of upregulation during the recovery period. Elevated oxidative stress in WATER compared with AIR is supported by the over-expression of Glutamate-cysteine ligase, the rate-limiting enzyme for glutathione synthesis, and by CYP49A, a cytochrome P450 involved in detoxification and defence.

Upregulation of several subunits of the T-complex (Table 1), TBCB (tubulin-folding cofactor B), EML1_2 (microtubule stabilization) and small muscle UNC-45 protein (a myosin folding chaperone required for cell shape and normal actin cytoskeleton) also suggest that WATER treatment caused more severe cytoskeletal disruption relative to AIR exposure. Furthermore, a range of genes with potential roles in immune, inflammatory and/or apoptotic processes were upregulated following high temperature exposure in WATER but not AIR. These included the increased expression of two DNAJ3 family members, which are involved in apoptotic signal transduction in mitochondria (Table 1). Caspase-domain proteins, several BIR-domain proteins (inhibitor of apoptosis family), and their downstream E3 ubiquitin transferases suggest activation/suppression of the apoptosis process, respectively (Table 1). Finally, different accessions annotated as SAMD9 (A0A6J8A7E0_MYTCO and A0A6J8DRH1_MYTCO), a tumor suppressor that may act in inflammatory responses to tissue injury, were upregulated in both lineages, although interestingly one (UniProt entry A0A6J8A7E0_MYTCO) was overexpressed only in the eastern and was downregulated in the western lineage (Table S3). Immune system-related transcripts were also overexpressed after exposure in WATER (but not AIR), including a Toll-like receptor (TLR3) and Peptidoglycan recognition protein (PGRP) (Table 1; see also Fig. 7ab and Table S3).
